# Patient-Reported Experience (PREMs) and Outcome (PROMs) Measures in Diabetic Foot Disease Management—A Scoping Review

**DOI:** 10.3390/jcm14176116

**Published:** 2025-08-29

**Authors:** Elisa Amato, Francesco Giangreco, Elisabetta Iacopi, Alberto Piaggesi

**Affiliations:** Diabetic Foot Section—Department of Endocrinology and Metabolism, University of Pisa, 56122 Pisa, Italy; elisa.amato92@gmail.com (E.A.); france.giangre@gmail.com (F.G.); elisabettaiacopi@gmail.com (E.I.)

**Keywords:** diabetic foot, foot ulcer, diabetes, self-perception, quality of life, quality of healthcare, outcomes

## Abstract

**Background/Objectives**: Diabetic foot syndrome (DFS) is a chronic complication of diabetes mellitus that negatively impacts patients’ quality of life (QoL). Patients’ perceptions of health status and healthcare can be assessed using Patient-Reported Experience Measures (PREMs) and Patient-Reported Outcome Measures (PROMs). This article aims to review the available literature on PREMs and PROMs, evaluate their characteristics, and determine whether an existing measure is applicable to or can be developed for the patient population of a third-level unit for diabetic foot. **Methods**: A search through Cinahl, Scopus, and Pubmed electronic databases was conducted to identify studies published between 2000 and 2024. Eligible studies included those using PREMs and PROMs in patients with DFS. Studies that used self-assessment methods or open questions and those that applied PROMs in people living with diabetes without diabetic foot were excluded. **Results**: After a careful selection, 53 studies met the inclusion criteria: none of these applied PREMs. Regarding PROMs, 46 studies applied a generic method alone or in combination with a specific tool to large populations comparing patient groups, while 7 studies applied a specific PROM alone to small populations evaluating specific aspects of pathology. **Conclusions:** In the existing literature, generic tools are mainly reported. No gold standard has yet been identified among all the tools considered for assessment of quality of life or patients’ perceptions of their health. Further studies are needed to develop a reliable and specific PREM or PROM questionnaire for complex patients affected by DFS.

## 1. Introduction

Diabetic foot syndrome represents one of the most severe chronic complications of diabetes mellitus and is defined by the International Working Group on the Diabetic Foot (IWGDF) as “the presence in the foot of a person with diabetes mellitus of one or more conditions such as peripheral neuropathy, peripheral arterial disease, infection, ulceration, Charcot neuro-osteoarthropathy, gangrene, or amputation” [1].

Among these conditions, diabetic foot ulcers (DFUs) have a significant social impact in terms of individual disability, hospitalizations, and public healthcare costs. It is estimated that annually between 7% and 20% of total medical expenses related to diabetes in North America and Europe are attributable to diabetic foot conditions [2,3,4].

Approximately one-third of the general population living with diabetes will develop a foot ulcer at least once in their lifetime, and over 50% of these individuals are at risk of developing an infection, increasing the likelihood of amputation [5]. Patients with DFUs have a 2.5 times higher mortality rate compared to people living with diabetes without ulcers and an estimated life expectancy that is reduced by 14 years compared to the general population [6].

Therefore, diabetic foot syndrome represents an important public health issue with significant negative consequences for healthcare systems and public health economics, as well as for patients’ quality of life [7].

Quality of life is an important aspect of the disease experience and influences how patients cope with the imposed conditions [8]. The World Health Organization (WHO) defines quality of life (QoL) as “an individual’s perception of their position in life within the cultural and value systems in which they live, and in relation to their goals, expectations, standards, and concerns”. It is a concept influenced by physical health, psychological state, level of independence, social relationships, and their interactions with environmental features [9].

In the context of disease, the term Health-Related Quality of Life (HRQoL) refers to a subjective measure of physical and psychological well-being, representing the patient’s own evaluation of how a disease affects their life [10].

Since the 1990s, research has increasingly focused on QoL as an essential health outcome, especially in chronic conditions, where active patient participation in the care process is required. This includes integrating the patient’s perspective and experiences and assessing psychological aspects, satisfaction with treatment, and overall well-being throughout the course of the disease [11].

Tools known as Patient-Reported Experience Measures (PREMs) and Patient-Reported Outcome Measures (PROMs) have been adopted to evaluate quality of life in a variety of health-related conditions and pathologies. These must be completed independently by the patient, and their use in clinical practice facilitates the planning of patient-centred care pathways [12,13].

PREMs provide a measure of the patient’s personal perception of the healthcare received during the treatment pathway. They help identify areas needing improvement in the patient experience, assessing healthcare staff engagement, in enhancing service quality, collecting data to inform the development and implementation of quality improvement initiatives, and for public reporting [14].

Patients experience related data can be collected via paper questionnaires delivered in person or by e-mail, electronic devices (e.g., tablets), online surveys, or through interviews or focus groups [15].

PROMs are standardized questionnaires used to quantify and qualify the patient’s perspective and perception of disability, functionality, and overall health status. They monitor the effectiveness and impact of interventions and treatments on health and quality of life during the care pathway or disease progression [13]. PROMs can measure various outcomes, including physical functioning, symptoms, overall health ratings, psychological and social well-being, cognitive functioning, role activities, personal constructs, satisfaction with care, HRQoL, and treatment adherence [12].

PROMs are classified as either generic or disease-specific; generic PROMs cover various aspects of health and disease outcomes and are applicable to broad and heterogeneous patient samples, allowing comparisons between groups with and without a condition [12]. However, due to their non-specificity, they may underestimate QoL changes in specific populations [7]. Commonly used generic PROMs include the EuroQol-5D Health Utility Index (EQ-5D), often used for cost-effectiveness assessments, the Medical Outcomes Study 36-item Short-Form Health Survey (SF-36) and its shorter version SF-12, and the RAND-36 developed by the RAND Corporation [10,16,17]. Another generic tool is the WHOQOL-BREF, the short version of the WHO Quality of Life assessment [9]. Disease-specific PROMs, on the other hand, provide additional and complementary information on patients’ QoL concerning a specific condition [7].

Although various condition-specific PREMs and PROMs are available in literature, no single tool is currently recognized as the “gold standard” for HRQoL assessment in patients with diabetic foot syndrome [10].

With the aim of adopting or developing specific PREMs and PROMs that could be administered to selected patients with diabetic foot syndrome in a third-level specialistic unit, we reviewed the literature to evaluate the available evidence and compare their characteristics.

## 2. Methods

We extensively searched the literature using the databases Cinahl, Scopus, and PubMed and the keywords “diabetic foot”, “foot ulcer”, “quality of life/health related quality of life”, “quality of care”, “social factors”, and “outcomes”, together with the operators “OR” and “AND”. For the keyword “diabetic foot”, the following subheadings were selected: complications, economics, epidemiology, ethnology, aetiology, mortality, nursing, pathology, prevention and control, rehabilitation, psychology.

The search was restricted to English language articles and those conducted on human populations.

The search included all papers published from 2000 to 2024 and bibliographies of relevant citations were screened for further articles of relevance.

Additional filters applied concerning article type included case reports, comparative studies, meta-analyses, multicenter studies, observational studies, randomized controlled trials, reviews, and systematic reviews.

Inclusion criteria specified that studies had to assess HRQOL in patients with diabetes (type 1/2) and foot problems using structured PROMs or PREMs. Studies using PROMs to look at aspects of HRQOL in populations living with diabetes without foot concern were excluded, as were studies that used ‘self-evaluation’ (i.e., free text/interviews) for evaluating HRQOL.

It should be noted that some of the studies considered also used specific PROMs for assessment of various aspects of patients’ lives (e.g., anxiety/depression). For this review, only those that take into account aspects related to the foot have been considered and reported because the other tools are beyond the scope of this review. This literature review was conducted in accordance with Preferred Reporting Items for Systematic Reviews and Meta-analyses (PRISMA) 2020 statement [18].

In the Supplementary Materials file have been reported iunventories and instruments adopted in many of the papers analysed in this review, available in PDF format.

## 3. Results

**Figure 1:**
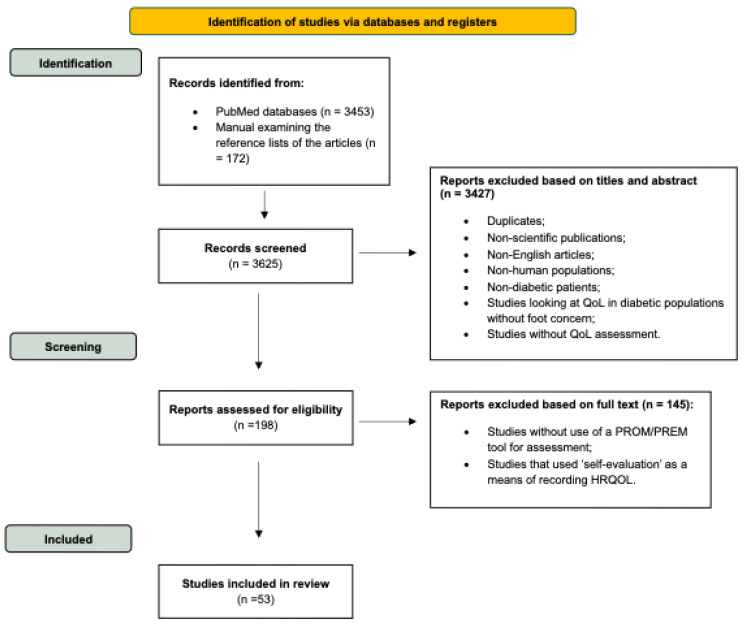
PRISMA 2020 flow diagram.

The initial search provided a total of 3625 articles. Of these, 3453 articles were retrieved trough databases. An additional 172 articles were identified by analysing the references lists of articles included. A total of 198 articles were selected and assessed for eligibility and subsequently 53 articles were included in this review. Figure 1 shows the PRISMA flow diagram for the studies included in this review.

From this literature review it can be observed that the application of PREMs tools in the diabetic foot field is lacking. In contrast, various generic and specific PROMs are used to assess HRQL in people living with diabetes with diabetic foot syndrome (Table 1 and Table 2).

Looking at previously reported tables (Table 1 and Table 2), generic PROMs were used most in the studies examined, with thirty-three studies using SF-36 and its shorter and adapted form (SF-12, SF-8, RAND-36) [20,21,22,23,24,25,26,27,28,29,30,31,32,33,34,35,36,40,41,43,44,45,47,52,53,54,55,56,59,62,63,66,68], seven studies using EQ-5D [19,37,42,49,51,60,69] and six studies using WHOQOL-BREF [8,38,39,48,57,65]. Ten studies used SF-36 and its shorter and adapted forms in association with specific PROMs like the Cardiff Wound Impact Schedule (CWIS) [24], the Diabetic Foot Ulcer Scale (DFS) and its shorter version DFS-SF [22,25,44,59], particularly in those studies that evaluate the impact of diabetic foot ulcer on patients’ QoL. SF-36 is frequently associated with the Foot and Ankle Ability Measure (FAAM) [41,52,53,56] and the association between SF-36 and FAAM is also noted in studies evaluating QoL in patients with Charcot neuroarthropathy [40,43] where considered populations are small, generally under one hundred patients. EQ-5D is used individually in studies with large population sizes [19,37,42,49,51,69] and in only one of the selected studies was NeuroQol used in association with a specific PROM [60]. WHOQOL-BREF has always been used singly in studies considering psychosocial characteristics and determinants [8,38,39,57,65] or assessing QoL in populations residing in specific and limited areas [8,48]. Seven studies use only a specific PROM for QoL assessment, specifically NeuroQoL [46], DFS [67], DFS-SF [50,58,70], CWIS [61], and WoundQoL [64].

In general, it can be observed that studies applying generic PROMs singly or in association provide comparisons between different patient groups. Studies using only specific PROMs for QoL assessment in patients with diabetic foot syndrome [46,50,58,61,64,67,70] consider small population samples have evaluated specific aspects or determinants that influence patients’ QoL and do not provide comparisons between groups of patients.

Only one of the selected studies is a randomized control trial (RCT) [60]; the others were observational studies. Among these, twenty-four were cross sectional, fifteen were prospective, eleven were case/control, one was retrospective, and one preliminary.

## 4. Discussion

This review was conducted to provide knowledge of which PREMs and PROMs had been used in previous studies to assess QoL in diabetic foot patients, and among these, which could be applied in a population of patients with diabetic foot in a context of a third-level centre.

The results showed a complete lack of PREM questionnaires applicable to patients with diabetic foot. PROMs have been used in a generic or specific fashion, often combined to explore more aspects of the disease and provide detailed information regarding specific outcomes.

The studies included in the review explore diabetic foot patients’ QoL mainly in relation to the presence of an ulcer, and the impact that it and the possible related consequences such as infection, non-healing, recurrence, or amputation have on all aspects of patients’ lives, both psycho-physical and social, and those of their caregivers. The potential negative impact of complications on QoL, including neuropathy in its most severe form of Charcot neuroarthropathy (CN), is also considered, as well as how some social, psychological, demographic, and geographical determinants and some clinical and gender characteristics can influence lesion management, disease acceptance, patient compliance, and adherence to treatment. In general, it can be stated that the presence of a diabetic foot ulcer, especially if associated with pain and infection, negatively affects all aspects of a patient’s QoL and that of their caregivers. It limits their physical functionality, physical and mental well-being, and social participation, with an impact also from an economic point of view. In patients with Charcot neuroarthropathy, QoL is reduced in terms of physical functionality, but there does not seem to be a significant impact of this complication on the psychological sphere; these findings may be due to bias related to the type of PROMs used and the method of calculating the score. Changes in lifestyle imposed by the pathology and its complications can determine the onset of anxiety and depression disorders, especially in patients with a first ulcer and in association with the patient’s age (<50 years), female gender, low level of education, and ulcer persistence (>7 months). The importance of psychological support for the patient and its positive impact on QoL throughout the entire course of the disease is highlighted. Improvement in quality of life in terms of physical function and psychological well-being is observed in patients whose ulcers heal or in those who have undergone minor amputation as they stop using dressings and offloading devices and can return to their daily activities while maintaining independence in walking.

The finding of a complete lack of PREMs is not surprising since there is a scarcity of this type of tools for the population living with diabetes. In a 2021 review, Martin-Delgado et al. identified only two specific PREMs for type 1–2 people living with diabetes [11]. Regarding PROMs, a number of instruments, both generic and specific, are available, and validated for HRQL assessment in patients with diabetic foot syndrome, but none of these can be considered as a gold standard, since each of them has limitations and lacks the properties to be able to capture all the aspects of the pathology, when used individually. From the results, it can be observed that the generic SF-36 is the most utilized PROM, used in twenty-nine studies [20,21,22,23,25,26,27,28,29,30,31,32,33,34,35,36,40,41,43,45,47,52,53,54,55,56,62,63,66]. It is often used to verify construct validity of a specific PROM; it has been used to distinguish among patients with varying degrees of diabetes severity, and between patients with or without complications of diabetes [71,72,75]. Studies conducted on large populations and clinical trials have demonstrated that SF-36 is useful for documenting differences between sick and healthy patients and for assessing the burden of different medical conditions [78]. SF-36 shows good reliability, validity, and psychometric properties and is the most used internationally for quality of life assessment, but it is a generic PROM, so its scores could be influenced by other complications of diabetes not related to the foot, due to lack of specificity [26,27,41]. For this reason, SF-36 is often combined with another specific PROM, to give more detailed information on specific outcomes [22,24,25,40,41,43,52,53,56]. SF-36 is also available in shorter versions, like SF- 8 and SF-12, that in turn can be converted in a six-items version, the Medical Outcomes Study 6D Short-Form Health Utility index (SF-6), and it is applicable for economic analysis [10]. The shorter versions measure the same eight domains as SF-36 and can be useful in clinical studies when time is short and patient burden must be considered [24,59]. An adaptation of the SF-36 questionnaire is the RAND-36, a tool with the same items and dimensions of the SF-36, and the same two summary scores deriving from the considered dimensions; the differences between the two questionnaires are in the score calculation and in the items’ coding, which is easier for RAND-36 in which the answers given to the items are averaged in the same scale together [17,55,77]. Another difference is in the treatment of missing data. In SF-36, this is handled by replacing the data that the patient did not provide with the mean of the scores of the same dimension, whereas in RAND-36, missing data of the items are not filled, since the mean is used to calculate the score for each dimension [17]. The RAND-36 is valid to assess HRQoL between patients with different diseases, over time, and to compare the burden of disease and its relative aspects in patients with chronic illnesses like diabetes and diabetic foot ulcer, for example [44,68,77]. Other generic tools for QoL assessment are the EuroQoL 5D Health utility index (EQ-5D), used in seven studies considered for this review [19,37,42,49,51,60,69], and the WHOQOL-BREF, used in six studies [8,38,39,48,57,65]. EQ-5D can be applied to describe and evaluate a treatment in economic terms, and is accompanied by a visual analogue scale (VAS 0–100) where patients indicate the perceived health state on that day [74]. The EQ-5D is easy to use because is short and is appropriate for both clinical outcomes and health economics evaluation [19]; it has also been shown to be sensitive to changes in health status after podiatric surgery and in patients with diabetic foot disease and foot ulcer, with an improvement in QoL scores in patients whose ulcer healed [51]; however, it is an elementary instrument, so it needs a large sample size for consistent results [37]. The WHOQOL-BREF is an instrument for subjective assessment of the global QoL, sensitive to the demographic characteristics of QoL and is designed as a multi-dimensional profile, enabling a wide range of diseases and conditions to be compared [9,82]. It has demonstrated good criteria, discriminant and content validity, and test-retest reliability with excellent internal consistency for all domains [83]. It is suitable for use in a clinical setting but also in clinical trials, when a brief assessment of QoL is requested; it is longer than other short form questionnaires like SF-12, for example, but it considers domains such as social relationships and environment that are not always evaluated in other questionnaires [83].

Specific PROMs to assess QoL in patients with diabetic foot problems and utilized in selected studies include Diabetes Foot Ulcer Scale (DFS) [22,25,67] and its short form (DFS-SF) [44,50,58,59,70], NeuroQoL [46,60], the Cardiff Wound Impact Schedule (CWIS) [24,61], Wound-QoL [64], and the Foot and Ankle Ability Measure (FAAM) [40,41,43,52,53,56]. In a 2012 review, Hogg et al. analysed 53 studies examining both generic and disease-specific PROMs used to assess QoL in patients with diabetic foot and identified the Diabetic Foot Ulcer Scale (DFS) and NeuroQoL as the most validated condition-specific PROMs [10]. DFS has shown the ability to distinguish between healed or unhealed ulcers and is sensitive to variations in ulcer status [72]. NeuroQoL has shown validity and reliability in evaluating the consequences of diabetic peripheral neuropathy on patients’ quality of life and it has shown a strong association with the clinical symptoms of neuropathy and their influence on global QoL compared to the generic SF-12 [76]. Romero-Collado et al. also recommend NeuroQoL, along with the Cardiff Wound Impact Schedule (CWIS), Norfolk QoL-DN, and Wound-QoL [7]. The CWIS is appropriate for the evaluation of patients with chronic wounds, showing internal consistency, good reproducibility, and good correlation with the generic SF-36; it can also distinguish between health states, discriminating the impact of wound on patient’s QoL from other concomitant factors [71]. Wound-QoL is the most practical in clinical settings due to its brevity (seventeen items in one page), comprehensibility, coherence and ease of administration [81]. Ortega-Avila et al. identified the Foot Health Status Questionnaire (FHSQ) as the most appropriate for evaluating patients with diabetes-related foot and ankle disease, while the most frequently used tool is the Foot and Ankle Ability Measure (FAAM) [84]. The FAAM has shown sensitivity to changes in foot and ankle functionality and is a region-specific questionnaire, applicable in various type of disorder but without specific items related to diabetes, so it would be appropriate for use in association with a disease specific instrument [75].

Another systematic review, published in 2021, identified the Diabetic Foot Self-care Questionnaire of the University of Malaga (DFSQ-UMA) and the Questionnaire for Diabetes-Related Foot Disease (Q-DFD) as appropriate tools, given their strong measurement properties. These two brief questionnaires, comprising 28 items in total, are suitable for combined use in evaluating patients with diabetic foot syndrome [85]. In 2024, Ruiz-Muñoz et al. developed and validated the Diabetic Foot Questionnaire (DiaFootQ), aiming to provide insights into foot health awareness, lifestyle habits, footwear, and disability outcomes. It considers modifiable disease-specific factors (e.g., footwear, lifestyle) and assesses the disease’s impact on patients’ daily routines [5]. The DiaFootQ consists of 25 questions across two domains lifestyle/function and footwear/foot self-care and isan acceptable model fit with good reliability for detecting changes in patients with diabetic foot syndrome [5].

The analysis of the literature has shown that diabetic foot syndrome, particularly when complicated by the presence of ulcers has a significant negative impact on patients’ QoL. Data show that there are some factors such as patient age, type of diabetes, and the depth and extent of the lesion and pain, that are related to a general reduction of QoL in diabetic foot patients, limit daily activities, reduce mobility, and worsen symptoms of anxiety and depression [25,35,48,54,66]. Other determinants include educational level, with a higher level contributing to a better understanding of the disease state and of information relating to the therapeutic process, in addition to high BMI, high levels of C-reactive protein (PCR) and glycated hemoglobin (HbA1C), and patient’s sex, with women showing lower HRQL scores than men, although young men are more likely to develop DFU [8,19,22,26,37,59,63,64,66,68]. Del Core et al. and Sanjari et al. reported that women had lower scores in two of the eight subscales of SF-36 (physical function and bodily pain), with the hypothesis that women have a better understanding of the pathology and its impact on their lives, have an active role in the general disease management, and are aware of the loss of their role within the home environment [34,56]. The aspects most significantly affected by the pathology that leading to a reduction in overall QoL include physical functioning, social participation, and psychological and mental well-being. Patients with active ulcers and a history of previous amputations reported a reduction in QoL scores in terms of daily activities, leisure time, anxiety, and social isolation [61,70]. In patients with diabetic foot ulcer who report low baseline QoL scores in the physical domains (mobility, self-care, usual activities, and pain/discomfort), a correlation with increased major amputations and death is observed [42]. Reduction in mobility is associated with the pathology and the limitations of deambulation due to the need to offload the lesion in order to improve ulcer healing, resulting in difficulty or inability to walk, climb stairs, or stand without the use of assistive devices or offloading orthoses [27,37,67]. Moreover, the use of a walking aid is associated with a score reduction in both the physical and mental domains of the SF-36, as it is synonymous of mobility reduction and balance impairment and seems to be a predictive factor for plantar foot ulceration in patients at high risk of ulcer development [66]. In the presence of peripheral neuropathy, balance and mobility problems worsen, further reducing physical functionality; if the neuropathy is associated with acute pain, cramps and burning sensation, the overall QoL and the psychological component score are reduced, which also occurs in the initial phases of disease progression [31,33]. Crews et al. showed that in patients with active ulcers, postural instability due to peripheral neuropathy is associated with a reduction in offloading adherence [46]. The presence of an ulcer and the associated burden significantly reduce the quality of life of patients and their caregivers: the longer the duration of the lesion (especially if it is over 12 months), the worse the QoL, particularly in terms of physical health, as well as treatment-related and economic burden and psychological well-being [22,23,45]. Polikandrioti et al. observed that physical limitation, social isolation and pain determined a reduction in the psychological well-being of patients with DFU, making them more prone to the onset of anxiety disorders and depression [62]. Pedras et al. also found high levels of anxiety and depression in hospitalized patients with DFU and indication for lower limb amputation [54]. Anxiety symptoms are more frequently observed in patients with diabetes duration of less than 10 years in the presence of comorbidities and glycated hemoglobin (HbA1c) values greater than 7%, while depressive symptoms are more associated with the age of the patient (<50 years), female gender, smoking habits, a low level of education, living alone, and ulcer persistence for more than 7 months; depressive symptoms are also associated with ulcer recurrence in patients older than 60 years [62]. Depression in patients with a first ulcer is associated with a two-fold increase in 5-year mortality and amputation rate; these patients also reported a greater reduction in overall QoL than those with recurrence, due to anxiety and concerns about the treatment process and outcomes [31,62]. Winkley et al. found that in patients with a first ulcer who experience the onset of other complications, scores in the physical functioning, general health, and mental health domains of SF-36 were reduced during 18 months of follow up, and the mental component score was further reduced in patients with non-healing or recurrent ulcer [30]. An improvement in QoL is observed in patients whose ulcers heal compared to those with chronic ulcers, especially in terms of physical functionality and healing, is also associated with an improvement in the psychological well-being of both the patient and the caregiver [23]. When patients heal, they resume their daily activities and can discontinue the use of offloading devices that limit mobility. The use of orthopedic footwear with custom-made insoles to cushion the foot and increase plantar contact area improves patients’ quality of life, with improvements observed in the physical and mental domains of the SF-36 as early as six months, along with a reduction in ulcer recurrence rates, as demonstrated by Davies et al. [20]. According to Spanos et al., ulcer healing was associated with the Texas wound classification and the infection score. Patients with lower-grade Texas wound classification had a higher probability of healing compared to those with higher-grade lesions; for each one-point increase in the infection score, the risk of ulcer non-healing increased by 15% [50]. Infected ulcers have the most impact on QoL reduction compared with healed, non-infected, hospitalised and amputated ulcer states, as shown by Byrnes et al., although all ulcerative conditions lead to a reduction in QoL [69]. A similar result was previously found by Raspovic et al., who reported a reduction in SF-36 physical and mental scores and FAAM scores in hospitalized patients with moderate to severe diabetic foot infections [41]. Goodridge et al. showed that non-healed ulcers can be associated with other concomitant pathologies that interfere with the ongoing treatment and worsen perceived physical health [24]. Raspovic et al. reported that end-stage renal disease has a significant negative impact on physical QoL in diabetic foot patients, without impact on mental quality of life [52]. In a study by Siersma et al. that investigated the effect of comorbidities on QoL during DFU treatment, they found an improvement in HRQL in patients whose ulcers healed, with no influence of comorbidities on this improvement for ulcers that healed within six months from baseline [49]. Also interesting is the result reported by Spanos et al., in which a significant improvement in the QoL of patients with DFU was observed in all DFS domains after 12 months of follow up regardless of the type of vascular treatment performed and the clinical outcome (healing or amputation) [50]. Iversen et al. studied the effect of a telemedicine intervention on DFU healing compared to standard of care and found that the EQ-5D score improved slightly in the standard of care group but not in the telemedicine group, and that the telemedicine intervention had no significant effect on ulcer treatment outcomes and did not result in worsening outcomes [60]. Pickwell et al. observed an improvement in quality of life in patients with a diabetic foot ulcer undergoing minor amputation, especially in scores regarding anxiety and depression of the EQ-5D questionnaire [51], while Pedras et al. found that mental QoL remained the same, with worsening physical QoL in patients with DFU before and after amputation surgery [47]. A similar result is provided by Juzwiszyn et al., who report that physical domain scores were worse than mental and environmental domains, with a positive correlation between QoL and acceptance of illness [65]. McDonald et al. found that amputation has a significant negative impact on body image disturbance, as an objective change in the patient’s body; while the low scores on measures of depression and physical QoL could have been due to the complicated general conditions of the study population [39]. Wukich et al. reported a QoL improvement in diabetic foot patients after trans tibial amputation, due to maintaining the ability to walk independently with a prosthesis after amputation [53]. A similar result was found by Boutoille et al. who showed lower scores of SF-36 for the group of patients with ulcer compared to the amputee group, especially for the item “bodily pain”, highlighting how amputation can improve the quality of life, especially when prosthetics are possible. This allows a faster rehabilitation and recovery of mobility [28]. Limitation in mobility is one of the most significant factors affecting the perceived quality of life (QoL) in patients with diabetic foot syndrome, particularly in the presence of foot-related complications such as Charcot neuroarthropathy (CN). Patients with Charcot neuroarthropathy report a decrease in QoL with regard to the physical functioning scores on the SF-36 and with a negative impact on lower limb function, as reflected in reduced FAAM scores, both in the presence and absence of ulcers, even in the long-term [21,29,40,43]. It is interesting to note that, in these patients, the overall score for the mental component is not significantly reduced compared to patients with diabetes mellitus without foot involvement [40,43]. A plausible explanation can be found in the fact that studies that take into account this type of population often use a generic questionnaire, such as the SF-36, to assess quality of life (QoL), which may lack the sensitivity required to capture the psychological impact of the disease, especially when mental component scores are calculated using orthogonal factor coefficients, which may underestimate mental QoL in patients with diabetic foot [40,43,55]. In this regard, Ahn et al., in their study, suggest calculating mental component scores using oblique factor coefficients, which are more sensitive to changes in mental QoL in patients with physical functional limitations due to diabetic foot and its complications [55]. The need for assistance due to the presence of a diabetic foot ulcer and the consequent mobility reduction, the high costs of health care, and the limitation of working activities have impacts on economic aspects of the patient’s life, while tendency to isolation and self-marginalization limits participation in social dynamics, leading to a lack of confidence in social interactions [32,38,48,67]. These aspects highlight the importance of social support in psychological resilience for patients who experience a DFU, with a positive effect on both QoL and self-management. In fact, patients who face the condition with the support of family or a community report higher QoL scores [57,58,63]. Ali Alzahrani et al. showed that the involvement in a spiritual community had a positive effect on mental QoL improvement in diabetic foot ulcer patients, especially in elderly patients, because religious connectedness can represent a coping mechanism with an helpful role in QoL improvement and in socialization [36].

This study has several limitations, mainly associated to the manual search and selection of included articles, despite extensive. Furthermore, other limitations are associated to the use of generic patient-reported outcome measures (PROMs) in most of the included studies, the lack of studies using patient-reported experience measures (PREMs) tools, and the small sample sizes in some of the studies considered, which may reduce the statistical power and generalizability of the findings.

## 5. Conclusions

Diabetic foot syndrome has a significant negative impact on patients’ QoL, who reported low and poor levels of QoL from both a physical and psychological perspective. This review has shown that many tools are available to assess QoL in patients with diabetic foot syndrome, and although none of these can be defined as the gold standard for the evaluation of these complicated patients. PREMs and PROMs tools are useful for evaluating chronic disease care pathways, and for understanding patients’ point of view on their health, their well-being, and their care experiences. In the studies considered, generic PROMs like SF-36, EQ-5D and WHOQOL-BREF are mostly used. These questionnaires are useful for use on large populations but are not able to evaluate and highlight all the pathological and psycho-social factors that influence the diabetic foot; this can lead to the risk of underestimating some aspects of the pathology (e.g., stress-related or psychological components). Despite this, the use of generic tools can be useful when a wide range of comorbidities occur simultaneously, as in most patients with diabetic foot. Unlike PROMs, which have been widely developed and used in clinical practice in recent years, the use of PREMs remains limited. There is a lack of applicable PREMs tools for the evaluation of patient experience of diabetic foot syndrome. Therefore, considering the observed results, we believe that this review highlights the need and lays the foundation for developing a PREMs questionnaire to assess the patient experience with diabetic foot syndrome throughout the healthcare pathway, from the first access to the diabetic foot clinic until healing, and beginning from extensive interview with patients [86]. It may also be useful to develop a PROMs questionnaire in parallel, with the aim to assess the overall aspects of diabetic foot disease and its impact on patient’s quality of life. This tool should address the limitations of currently used PROMs, which tend to underestimate some aspects of the pathology. In both cases, the aim would be to develop tools that might be useful in daily clinical practice and applicable for research purposes; these tools will have to consider the workload of healthcare professionals, as well the patient’s burden.

## Figures and Tables

**Table 1:** Selected Studies in chronological order.

Reference	Study Design	Study Aims	Study Results	PROMs Used
Ragnarson Tenvall, Apelqvist Sweden, 2000 [19]	Cross-sectional 310 patients, 4 groups	To assess QoL in people living with diabetes with ulcer, with healed ulcer and in those with amputation.	Current ulcers and major amputation have a negative impact on QoL and reduce EQ-5D index compared to patients who heal primarily without amputations or who undergo minor amputations.	EQ-5D-3L
Davies et al. U.K, 2000 [20]	Case-control 280 patients, 4 groups	To compare the health status of a group of people living with diabetes receiving orthotic therapy with a group not receiving it.	Orthotic therapy resulted in a statistically significant increase in all SF-36 scores, both physical and mental, at 6- and 12-month assessment compared to the comparison group in which a worsening of QoL was observed	SF-36
Willrich et al. USA, 2005 [21]	Preliminar—pilot study 60 patients, 3 groups	To determine if there is a correlation between podalic complications such as DFU, infections, NOA (neuro-osteo-arthropathy), amputations and cognitive impairment or depression.	DFU, Charcot NOA, and amputation have significant negative impact on QoL at SF-36 scores without demonstration of cognitive impairment or depression.	SF-36
Valensi et al. France, 2005 [22]	Cross-sectional- observational 355 patients, 2groups	To compare QoL in DM patients with/without DFU and determine specific pathology factors influencing QoL in patients with DFU.	DFU patients have lower SF-36 scores compared to those without foot ulcers. Worse DFS scores are found for higher Wagner grades, long-standing ulcers, and multiple ulcers. Age is associated with lower DFS scores for the domains of daily activity and dependence of DFS.	SF-36 DFS
Nabuurs-Franssen et al. Holland, 2005 [23]	Prospective-multicenter 294 patients and 153 caregivers	To determine the impact of ulcer healing on the QoL of people living with diabetes and their caregivers.	Patients with healing ulcers have higher QoL scores than patients with persistent ulcers both 20 weeks after baseline (T1) and 12 weeks after T1 (T2), particularly for the SF-36 domains of physical and social function, physical role, and the physical summary score. The caregiver scores of the emotional role subscale improve for those of patients who heal compared to those for whom the ulcer persists, especially in T2.	SF-36
Goodridge et al. Canada, 2006 [24]	Cross-sectional- comparative 104 patients, 2 groups	Evaluate QoL in patients with healed and non-healed DFUs.	Patients with non-healed ulcers have lower PCS (physical component summary score) score on SF-12 than those with healed ulcer. No differences are observed for MCS (mental component summary score) score between the two groups. Regarding the CWIS (Cardiff wound impact schedule) scores, patients with non-healed ulcers result frustrated and anxious with low well-being component score.	SF-12 CWIS
Ribu et al. Norway, 2006 [25]	Observational 127 DFU patients	To describe the prevalence and occurrence of DFU-associated pain and the impact on HRQL using generic and disease-specific instruments.	75% of DFU patients’ sample have pain during walking/standing and/or in the night. Patients who reported DFU related pain show low score in all DFS and SF-36 subscales.	SF-36 DFS (Diabetic Foot ulcer scale)
Ribu et al. Norway, 2007 [26]	Cross-sectional 127 DFU patients	To describe sociodemographic variables, clinical characteristics, and treatment factors in patients with diabetic foot ulcer and explore the association between these factors and patients’ QOL.	Clinical variables with a negative effect on SF-36 score are BMI (body mass index) < 25 kg/m^2^, nephropathy, an ABPI (ankle brachial pressure index) value < 0.9, CRP (C reactive protein) levels > 10 mg/L and ulcer size greater than 5 cm^2^. No significant influence of demographic variables on QoL was found in the selected patient sample.	SF-36
Ribu et al. Norway, 2007 [27]	Cross-sectional 127 DFU patients—221 DM patients—5903 general population	To describe HRQL in patients with DFU by comparing their HRQL with that of a general population sample without diabetes and a subgroup with diabetes and to examine differences between groups based on sociodemographic characteristics and lifestyle factors.	DFU patients scored significantly lower in all SF-36 domains compared to the population living with diabetes samples and also compared to the general population sample. BMI was higher for DFU patients compared to the other population samples and most DFU patients lived alone compared to the other two population samples.	SF-36
Boutoille et al. France, 2008 [28]	Case-control-retrospective 34 patients, 2 groups	Understanding the influence of amputation on the physical and social aspects of QoL in patients with diabetic foot.	Patients with DFU reported lower score for the items “Bodily pain” and “Role Physical” of SF-36 than the amputation group. The global scores of SF-36 were poor in both groups.	SF-36
Pakarinen et al. Finland, 2009 [29]	Cross-sectional-follow up 29 patients	To evaluate long-term effects and QoL of patients with Charcot after a minimum of 5 years of follow-up.	67% of patients with Charcot foot had one ulcer during the follow up period and 40% of patients had an ulcer once or more. 50% of patients underwent surgery. The functional outcome was better for patients who were diagnosed within 3 months. Patients with Charcot foot reported lower SF-36 scores compared to general population and to chronically ill population, particularly for role physical, role emotional and social functioning domains; These scores are reduced for patients with walking ability of less than 500 m.	SF 36
Winkley et al. U.K., 2009 [30]	Cohort-Prospective 253 patients	To prospectively describe changes over 18 months in QoL of patients with DM and newly discovered ulcer.	At 18-months FU (follow up) patients with a first DFU reported a decrease in SF-36 scores for physical functioning, mental health and general health domains. A significant decrease in MCS scores was observed in patients who did not heal and in those who had recurrence of ulcers.-Non-significant reduction was observed in amputated patients.	SF-36
Garcia-Morales et al. Spain, 2011 [31]	Comparative-prospective 421 patients, 2 groups	To determine the impact of etiological and pathogenetic factors of diabetic foot on various aspects of QoL in the Spanish region of Gran Canaria.	A statistically significant difference in the overall score of SF-36 between group 1 (no ulcer) and group 2 (ulcer); the lower scores for group 2 were recorded in physical functions, physical role limitation and vitality domains. Neuropathy and poor metabolic control significantly reduce patients’ QoL. Patients with an ulcer for less than 2 months had higher QOL than those with a lesion for more than 3.	SF-36
De Meneses et al. Brasil, 2011 [32]	Cross-sectional comparative 35 patients, 2 groups (DFU—NO DFU)	Assessing HRQoL and self-esteem in patients with DFU.	Patients with DFU had lower SF-36 scores than those without foot ulcer, particularly for physical functioning, social functioning, and emotional domains. No differences regarding self esteem between groups were observed. Women on average had a higher QOL score than men.	SF-36
Jelsness-Jørgensen et al. Norway, 2011 [33]	Cross-sectional 157 patients, 2 groups (DM no DFU—DM + DFU)	Describe HRQOL in DM patients without DFU and identify sociodemographic and/or clinical variables that significantly influence HRQOL and investigate DFU effects on HRQOL, comparing patients with and without DFU.	Patients with DFU have more comorbidities than those without, especially cardiovascular (6 times more at risk). Patients with diabetic foot ulcer had reduced scores in 7 out of 8 domains of SF-36 especially in physical domains.	SF-36
Sanjari et al. Iran, 2011 [34]	Cross-sectional 132 patients, 2 groups	To evaluate the factors influencing QoL of Iranian patients with DM and the effect of DFU on QoL in patients with DM.	DFU patients reported lower SF-36 scores in physical functioning, physical, bodily pain, social functioning, and emotional role domains than patients without DFU. Women had lower overall QoL, lower scores in physical functioning dimension and higher body pain.	SF-36
Yao et al. China, 2012 [35]	Observational perspective 131 patients DFU	To investigate the demographic, lesion and QoL characteristics of patients with DM at first visit for new ulcer, the influence of diabetic foot and its severity on the QoL of patients, and the influence of ulcer aetiology on the QoL of patients.	No statistically significant differences were observed between demographic and laboratory data for patients with different Wagner grade lesions. Patients with DFU had significantly lower scores in all domains of SF-36 than the general population. Patients with higher Wagner grade had lower SF-36 scores in all domains.	SF-36
Ali Alzahrani et al. Saudi Arabia, 2013 [36]	Observational, case-control 180 patients, 2 groups	Observe association between religious connection and QoL in patients with and without DFU.	DFU patients had reduced QoL in all SF-36 scores compared to people living with diabetes without lesions and healthy controls, and this reduction increased with ulcer severity, duration, and number. A positive relationship was observed between religious connectedness and MCS score for patients with DFU.	SF-36
Siersma et al. Europe, 2013 [37]	Prospective-observational Multicenter 1232 patients	To identify factors responsible for a reduction in QoL associated with DFU and their relative importance.	Patients with DFU had a reduction in overall QoL, with the primary determinant of reduction being the inability to stand or walk without help. Other factors that reduced the EQ-5D score included ulcer size, CRP concentration, and ischemia. Factors affecting pain/discomfort domain scores included infection, PAD (peripheral arterial disease), and DPN (diabetic peripheral neuropathy).	EQ-5D-3L
Fejfarova et al. Czech Republic-UK-USA, 2014 [38]	Case-control 152 patients, 2 groups	To evaluate the impact of diabetic foot on daily life, on psychological and socioeconomic aspects of patients and compare results with patients with DM without complications from DF.	DF (diabetic foot) patients have a worse QoL than controls without diabetic foot, especially in terms of physical health and environment, and are more frequently depressed. DF patients have basic education and, a small percentage work and are poorly self-sufficient. The presence of previous amputations has a negative impact on the environmental domain and employment status.	WHOQOL-BREF
McDonald et al. Australia, 2014 [39]	Case-control 270 patients, 2 groups: 50 amputees and 240 diabetics no amputation as controls	To statistically assess possible group differences on medical and demographic variables to examine the psychosocial impact of diabetes-related amputation.	The presence of amputation leads to medical problems such as increased insulin use, micro/macrovascular complications. Patients with diabetes and amputation have more depressive symptoms, poorer physical QoL, and greater disturbance of body image. After multivariate analysis, these differences remain for body image.	WHOQOL-BREF
Raspovic, Wukich USA, 2014 [40]	Case-control 106 patients, 2 groups	To compare QoL in patients with CN (Charcot neuro-osteoarthropathy) and in patients with DM without podalic involvement.	Patients with CN had lower FAAM (foot and ankle ability measure) scores than controls without foot involvement. For SF-36 scores, no differences in MCS were observed, while PCS was reduced compared to controls. Patients with CN had lower scores in 7 of 8 SF-36 domains.	SF-36 FAAM
Raspovic, Wukich USA, 2014 [41]	Case-control 47 patients with infection and 47 controls	To compare QoL between patients admitted for DFI (diabetic foot infection) and patients with DM (diabetes mellitus) without foot involvement.	Patients admitted with DFI reported low scores in all domains of SF-36, including PCS and MCS. FAAM scores were also significantly reduced compared to the control group without foot involvement (2 SD (standard deviation) on ADL (activity of daily living) score and 1.5 SD on SPORT score).	SF-36 FAAM
Siersma et al. Europe, 2014 [42]	Prospective-observational Multicenter 1015 patients	To evaluate whether QoL in patients with newly discovered ulcer has prognostic significance for healing, major amputation or death.	In patients with newly discovered ulcers, HRQL is not a predictor for healing, while a reduction in QoL scores in physical domains is associated with a significant increase in major amputations and death.	EQ-5D-3L
Raspovic et al. USA, 2015 [43]	Case-Control 57 patients, 2 groups	To compare QoL in patients with CN with a group of patients with CN and midfoot ulcer.	No significant differences were observed in SF-36 scores between patients with Charcot without ulcer (group 1) and those with ulcer (group 2) nor between FAAM scores. The only difference observed between the two groups was that patients with CN without ulcer had lower scores in the bodily pain domain than those with CN and ulcer.	SF-36 FAAM
Sekhar et al. India, 2015 [44]	Cross-sectional 400 patients, 2 groups	Evaluate QoL among people living with diabetes with and without ulcers.	DFU patients showed statistically significant differences compared to patients without DFU in all SF-36 subscales, especially physical functioning, role limitations due to physical health problems, and role limitations due to emotional health. A significant difference was also observed in MCS and PCS scores. For DFU patients, DFS-SF scores were low in all domains.	SF-36 DFS-SF (diabetic foot ulcer scale—short form)
Hoban et al. Canada, 2015 [45]	Case-Control 96 patients, 2 groups and 21 caregivers	To evaluate the effect of diabetic foot-related problems on mental well-being in patients and caregivers.	Diabetic foot patients reported lower scores in 6 of 8 SF-36 domains and in the PCS compared to those without foot involvement, were more depressed, andhad more pain and suicidal behaviours. Caregivers reported higher levels of anxiety and depression, and a positive correlation was observed between alcohol dependence and the MCS of SF-36 in caregivers of diabetic foot patients.	SF-36
Crews et al. U.K-USA, 2016 [46]	Prospective Multicenter 79 patients DFU	To evaluate the association between adherence to offloading and the number of ulcers healed during the treatment period. To identify potential physical and psychological determinants of adherence to offloading.	Smaller ulcers at baseline and better adherence to offloading significantly predicted reduction in ulcer size at 6 weeks. Larger and more severe ulcers at baseline, severe neuropathy, and higher NeuroQoL pain scores were significantly predictive of offloading adherence. Postural instability worsened offloading adherence.	NeuroQoL
Pedras et al. Portugal, 2016 [47]	Longitudinal 108 patients DFU	To identify predictors of HRQoL after surgery, to analyse the differences in HRQoL before and after surgery, to explore the moderating role of 1st vs. previous amputation in the relationship between physical and mental HRQoL before and after surgery in DFU patients.	SF-36 PCS score was significantly reduced after surgery, MCS score did not show significant differences. Previous amputation had a moderating effect on PCS and MCS scores before and after surgery.	SF-36
Nemcová et al. Slovakia, Czech Republic, Poland, Hungary 2017 [48]	Cross-sectional 525 patients DFU	Assessing QoL in patients with DFU in the Visegrad region.	Demographic determinants of QoL reduction were identified, including higher age, living alone, need for care, not belonging to groups or organizations. Clinical determinants of QoL reduction were high frequency of ulceration, higher severity of ulcer according to Wagner classification, presence of pain, high BMI, long duration of diabetes, and ulcer treatment. Significant differences in QoL were observed between the geographical regions considered in the study in all WHOQOL-BREF domains, with patients from Hungary having the worst scores in all domains.	WHOQOL-BREF
Siersma et al. Europe, 2017 [49]	Prospective observational cohort study 1232 patients	To evaluate whether the presence of diabetic complications also influences the improvement of HRQoL during DFU treatment.	Significant improvement in QoL is observed in almost all EQ-5D domains when the ulcer heals, regardless of whether it heals within 6 or 12 months. Baseline QoL is worse in the presence of comorbidities in patients who heal within 1 year and in those who do not heal, compared to those who heal within 6 months. In patients who do not heal after 12 months, the usual activities and pain/discomfort domain scores and EQ-5D index improve even in the presence of heart failure. An improvement in the usual activities score is also observed even in the presence of visual impairment or neurological disorders.	EQ-5D-3L
Spanos et al. Greece, 2017 [50]	Prospective non-randomized cohort study 103 DFU patients	To evaluate factors associated with the healing process or limb salvage, in patients with DFU and assess the impact of treatment on QoL.	Ulcer healing is associated with the Texas University Wound Classification grade and the degree of infection. Lower Texas grade ulcers are more likely to heal, and for every 1-unit increase of the infection score, the risk of non-healing increases by 15%. The likelihood of minor amputation increases for lesions grade > II of the Texas classification, in the presence of COPD (chronic obstructive pulmonary disease), and as the neuropathy score increases. The risk of major amputation is associated with a non-palpable popliteal artery, and hospitalizations, and increases by 3.5% for each day of delay until referral. QoL improved significantly in all DFS-SF domains after 12 months of follow up regardless of treatment type.	DFS-SF
Pickwell et al. Europe, 2017 [51]	Prospective-observational Multicenter 1088 patients, 2 groups	To determine the impact of minor amputations on QoL by comparing changes in QoL in patients healed after minor amputation with those in patients healed by primary intention.	Conservatively treated ulcers heal faster than those resulting from minor amputations, which are larger, deeper, often infected and localized to the toes. In patients who heal, QoL improves significantly. EQ-5D scores showed an improvement, although not significant, in patients who underwent minor amputations compared to conservative treatment.	EQ-5D-3L
Raspovic et al. USA,2017 [52]	Case-control 90 DFU patients, 2 groups 30 ESRD (end stage renal disease) and 60 no ESRD	To assess the impact of end-stage renal disease on QOL in patients with diabetic foot.	Patients with diabetic foot and ESRD had lower scores for the SF-36 physical, social and PCS domains than patients with diabetic foot without ESRD. No differences were observed between the two groups for the MCS score and the other SF-36 domains and for the FAAM score. Patients who underwent major amputation or surgery reported lower SF-36 scores in the PCS and physical, social, role-emotional domains. No significant impact on MCS was observed for mortality, amputation, or surgery. ESRD was significantly related to PCS score but not to MCS score.	SF 36 FAAM
Wukich et al. USA, 2017 [53]	Observational cohort study 81 patients	To evaluate HRQoL after major lower limb amputation in a cohort of patients with diabetes mellitus.	Patients who completed the questionnaires before and after transtibial amputation surgery showed significant improvement in all SF-36 domains, PCS and MCS, and FAAM scores. Post-intervention scores improved if the prosthesis allowed effective ambulation. Patients who were unable to ambulate pre-intervention had significantly lower scores on the SF-36 physical domains. Patients who were non-ambulant post-intervention had lower scores on the SF-36 general health domain and lower FAAM scores than those who were ambulant post-intervention.	SF 36 FAAM
Pedras et al. Portugal, 2018 [54]	Cross-sectional 202 patients DFU	To analyse the relationships between anxiety, depressive symptoms and functionality as predictors of QoL in patients with DFU, considering clinical variables.	A negative correlation was observed between PCS and MCS scores and gender, age, number of hospitalizations in the past year, pain, depression and anxiety symptoms. PCS score wss also negatively associated with ulcer duration. Negative predictor factors for MCS were anxiety and depression symptoms. Negative predictors for PCS were pain and depression symptoms.	SF36
Ahn et al. USA, 2018 [55]	Retrospective 300 DM patients, 2 groups	To assess physical and mental health-related QoL in patients with DM with or without diabetic foot and to evaluate whether mental health-related QoL is significantly different using orthogonal or oblique factor analysis.	Patients with diabetic foot complications showed significantly lower PCS scores when calculated with both orthogonal and oblique rotation factor coefficients. MCS was significantly reduced when calculated with oblique rotation factor coefficient.	SF36
Del Core et al. USA, 2018 [56]	Cross-sectional comparison 240 patients 120 M–120 F	To evaluate the impact of gender on QOL with a generic tool (SF 36) and a specific one (FAAM) in a cohort of male and female patients with diabetic foot.	Women showed significantly lower PCS scores than men, calculated with both orthogonal and oblique rotation coefficients. The worst scores were found in the domain of physical function and bodily pain. No significant differences are observed between men and women for MCS scores with both orthogonal and oblique rotation coefficients. No significant differences are observed between men and women for FAAM scores	SF36 FAAM
Alosaimi et al. Saudi Arabia, 2019 [57]	Case-control 209 DM patients: 45 DFU and 164 controls	To compare QOL and its psychosocial determinants between patients with and without diabetic foot ulcers.	There was a negative correlation with QoL and presence of symptoms of anxiety, depression and the severity of somatic symptoms. After multivariate analysis, depression remained a negative determinant of QoL, regardless of DFU status.	WHOQOL-BREF
Khunkaew et al. Thailand, 2019 [58]	Cross-sectional 41 DFU patients	Evaluating QoL and foot management in people with DFU.	The DFS-SF scores reported by the population considered are high and indicate good QoL. The lowest score was observed in the “worried about ulcers” domain. Only a third of patients reported having received education on foot care. Almost all patients (97.6%) wash their feet every day, most do not test the water temperature and approximately 63% dry between the toes; 70% of patients report walking barefoot at home. The most frequent barrier to foot care is not having a mirror to check the sole of the foot, and lack of knowledge on how to use it correctly.	DFS-SF
Alrub et al. Jordan, 2019 [59]	Cross-sectional 144 patients DFU	To determine factors associated with QoL among people living with diabetes with DFU in Jordan.	DFU patients reported low DFS-SF and SF-8 scores. Higher DFS-SF scores were associated with male gender, high level of education, no stressful events in the past year, not having PVD (peripheral vascular disease), no soft issue infection, lower Wagner classification grade, and normal BMI.	DFS-SF SF 8
Iversen et al. Norway, 2020 [60]	RCT 182 DFU patients 94 Telemedicine and 88 Outpatient	To compare changes in health, well-being, and QoL between patients with DFU undergoing telemedicine follow-up and patients undergoing standard outpatient care.	No significant differences were observed in the scores between the group of patients followed with SoC (standard of care) and that with telemedicine, which were almost unchanged between the baseline and follow-up measurements with both generic and specific PROMs. Telemedicine did not have significant effects on health, well-being and QOL outcomes compared to the SoC.	EQ-5D-5L NeuroQoL
Jayalakshmi et al. India, 2020 [61]	Cross-sectional, descriptive 118 DFU patients	To evaluate the impact of DFU on the different components of patients’ QoL and determine the associated factors.	Patients with DFU reported the lowest percentage of CWIS scores in the domain of “well-being” and the lowest in the domain of “social life stress”. A positive correlation was observed between QoL and the satisfaction domain of CWIS; a negative correlation was observed between QoL and satisfaction with stressful experience of social life and physical symptoms experience. The factors that most influence QoL are symptomatic living, social experiences, and stress, followed by satisfaction and then by stress in social life.	CWIS
Polikandrioti et al. Greece, 2020 [62]	Cross-sectional 195 patients DFU	To explore QoL levels in DFU patients and associated factors involved and the impact of anxiety, depression and treatment adherence on QoL.	Patients with DFU report moderate levels of QoL in the SF-36 general health domain and moderate to high levels in the SF-36 emotional well-being, pain, social functioning, and energy/fatigue domains. The lowest scores are reported in the physical functioning, role physical, and role emotional domains. Factors negatively associated with patients’ QoL and statistically significant are: age, education, number of children, other concomitant pathologies, daily blood glucose measurement, Wagner classification and alcohol consumption, as well as work and smoking habits but also compliance, with periodic checks and treatment and place of residence. QoL is better if anxiety and depression levels are low and based on adherence to exercise guidelines.	SF-36
Kuang et al. China, 2021 [63]	Cross-sectional, prospective 98 DFU patients	To determine the role of psychological resilience in the QoL of patients with DFU and in the regulation of self-efficacy, together with the risk factors of psychological resilience.	It is observed that patients with high psychological resilience had significantly higher levels of SF-36 for self-efficacy, general health, vitality, social functioning, role emotion, and mental health domains compared to participants with low psychological resilience. Negative determinants of self-efficacy are low psychological resilience, older age, low education, unemployment, and higher level of HbA1c. Negative determinants of QoL are low psychological resilience, older age, lower perceived social support, and higher level of HbA1c. Men have lower psychological resilience than women.	SF-36
Reinboldt-Jockenhöfer et al. Germany, 2021 [64]	Cross-sectional, Retrospective, Multicenter 381 patients (171 with DFU)	To investigate differences in physical, psychological and daily life-related QoL in patients with different causes of chronic wounds.	DFUs accounted for 44.8% (171/381) of the selected patient sample. Patients with DFU were significantly younger than those with arterial leg or mixed ulcers and were more frequently male. Patients with DFU reported discomfort in the “everyday life-related QoL” domain. The diagnosis of diabetic foot ulcer determined lower scores in the psychological subscale, a reduction in the “everyday life-related QoL” score and was a predictor variable for the reduction of general wound-related QoL. Females reported reduction in psychological and general wound-related QoL.	Wound-QoL
Kolarić et al. Croatia, 2022 [8]	Observational 382 patients with DM2 (diabetes mellitus 2)	To compare the QoL of patients with type 2 diabetes based on their chronic complications.	Patients with DM2 in the selected sample present neuropathy as the major complication, followed by retinopathy; nephropathy and DFU are represented in equal percentage. Patients with complications are older. Hospitalizations are more frequent in patients with DFU. Patients with DFU reported the highest WHOQOL-BREF scores in the psychological domain, as did patients with neuropathy, but the lowest in the physical domain. Low scores in the physical functioning domain are also reported by patients with nephropathy. Patients with neuropathy and retinopathy reported low scores in the social functioning domain. High scores were reported in the environmental domain by patients with nephropathy and retinopathy. Patients with multiple chronic complications report low scores for the physical functioning domain but high for the environmental domain.	WHOQOL-BREF
Juzwiszyn et al. Poland, 2022 [65]	Observational 99 patients	To evaluate the relationship between QOL, level of disease acceptance and nutritional status in diabetics undergoing lower limb amputation (58 major and 42 digital amputations).	Patients reported higher WHOQOL-BREF scores in the social domain, intermediate in the environmental and mental domains, and lower in the physical domain. Women reported lower scores than men, but no significant correlations were observed between gender and QoL; 5.1% of the patients considered are malnourished and statistically significant differences are observed between gender and age with nutritional status. Women have a worse nutritional status than men and older people are more malnourished. Less malnutrition corresponds to better QoL. Older patients accept the disease less. Patients with higher scores in all QoL domains and who report a better QoL accept the disease better.	WHOQOL-BREF
Perrin et al. Australia-Holland, 2022 [66]	Cross-sectional analysis of multicenter RCT (randomized control trial)s 304 patients	To evaluate QoL and associated factors in patients with DM at high ulcerative risk.	In patients at high risk of developing DFU, the highest score is observed in the mental health domain and the lowest for the general health domain on the SF-36 questionnaire. Factors associated with QoL are the use of walking aids with lower mean scores in the physical functioning, role physical and bodily pain domains, ethnicity with Caucasian patients having higher scores in the physical, emotional, mental health, social functioning, and bodily pain domains.	SF-36
Delpierre et al. U.K., 2023 [67]	Monocentric longitudinal observational 18 patients	To evaluate the short-term impact of non-removable offloading devices on physical activity and diabetic foot ulcer-related QOL in a small sample of community-dwelling people with DFUs.	After three weeks of follow-up, a significant difference in DFS score for non-compliance domain was observed. No differences in DFS scores were observed at six-week follow-up or at multiple time points. Patients reported low scores in the physical activity domain already at baseline, with worsening scores at 3 and 6 weeks in 2/3 of patients. The results suggest that non-removable offloading devices may have a negative effect on patients’ adherence to overall diabetes treatment and may impact financial well-being due to indirect costs, such as travel to periodic check-ups and reduced income due to reduced working capacity.	DFS
Hamid et al. Sudan, 2024 [68]	Cross-sectional comparison 120 patients, 2 groups	To compare QoL between people living with diabetes with and without DFU and determine factors related to low QoL.	DFU patients reported lower scores in 5 of 8 domains of SF-36 compared to diabetics without ulcer, but reported higher scores in the domains of general health perception and emotional well-being. There was a significant difference in the general score of SF-36 between the two groups, greater in the group of people living with diabetes without DFU. Women had lower scores than men in the domains of physical functioning and general health. Divorced or widowed patients reported lower scores, a higher level of education was a positive predictor of QoL especially in the domains of emotional role, energy/fatigue and pain. Ulcer duration correlated positively with all domains of SF-36 except the social one.	RAND-36
Byrnes et al. Australia, 2024 [69]	Cross-sectional multicenter 376 patients, 5 groups	Evaluate and compare QoL in patients with DFU: recovered, non-infected, infected, hospitalized and amputated.	75.3% of patients reported mobility problems, 35.6% self-care problems, 71.3% problems with daily activities, 69.7% pain/discomfort, 56.1% anxiety and depression. Patients admitted for DFU were younger than the other groups. Compared to patients with healed ulcer, those with non-infected DFU had more mobility problems while those with amputation were unable to perform daily activities. Patients with infected DFU had significantly lower EQ-5D index and VAS (visual analogue scale) scores than the other groups. The infected DFU condition remained the worst and maintained significant score differences compared to the healed ulcer status even after adjustment for sex and age.	EQ-5D-5L
Alvaro-Afonso et al. Spain, 2024 [70]	Cross-sectional observational 141 patients	Assessing QoL of Spanish Patients with DFU Using DFS-SF.	The lowest score was observed in the “worried about ulcers” domain of the DFS-SF questionnaire, while the highest was observed in the physical health domain. Regarding this domain, significantly lower scores were reported for patients with ischemic DFU. Significant differences between the physical health and “worried about ulcers” domains were observed, depending on level of education. Patients with dyslipidaemia reported lower scores in both the “worried about ulcers” and “bothered by ulcer care” domains. In the case of previous minor amputation, significant differences were observed in the domains of leisure, dependence/daily life, negative emotions, “worry about ulcers/feet”, and “worry about ulcer care”. A significant negative correlation was observed between the SINBAD (site, ischemia, neuropathy, bacterial infection, area, depth) classification score and the DFS-SF leisure, physical health, dependence/daily life, and “bothered by ulcer care” domains. A significant negative correlation was observed between the ulcer duration and all the DFS-SF domains.	DFS-SF

QoL: Quality of Life; DFU: Diabetic Foot Ulcers; CN/NOA: Charcot Neuroarthropathy/neuro-osteoarthropathy; PCS: Physical Summary Score; MCS: Mental Summary Score; BMI: Body Mass Index; ABPI: Ankle Brachial Pressure Index; FU: Follow Up; DM: Diabetes Mellitus; CRP: C-Reactive Protein; PAD: Peripheral Arterial Disease; DPN: Diabetic Polyneuropathy; DF: Diabetic Foot; DFI: Diabetic Foot Infection; COPD: Chronic Obstructive Pulmonary Disease; ESRD: End Stage Renal Disease; PVD: Peripheral Vascular Disease; SoC: Standard of Care.

**Table 2:** PROMs used in selected studies in alphabetical order.

Instrument	Type	Domain/Subscale	Scoring System	Comment	Studies Using PROM
CWIS Cardiff wound Impact Schedule [7,10,71]	Specific	26 questions in 3 domains: physical symptoms and daily life, social life, general well-being → 5-point Likert scale + 2 questions measuring global QoL and satisfaction with QoL. For these 2 items, questions are graded on a 11-point Likert scale	Score of each question is summed and transformed into a 0–100 scale Higher score = better QoL	For all chronic wounds Shows sensitivity to wound healing in RCTs evaluating type of dressing in DFUs. Lacks sensitivity to wound severity.	24; 61
DFS Diabetic Foot Ulcer Scale [10,72]	Specific	58 questions in 15 subscales (Leisure, Physical health, Daily life, Dependence, Family and friends, Treatment compliance, Positive relationships, financial burden, Side effects, Diet, Compliance, Medical complications, Satisfaction) Questions are graded on a 5-point Likert-type scale (1 = “not at all or none of the time”, 5 = “a lot, all of the time, or extremely”).	Score 0–100 (0 = poorer QoL)	Developed to assess the impact of DFU and treatment on QoL in people with diabetes. Specific measure for DFU and not generic for diabetes. Ability to discriminate between patients with diabetes and healed ulcer or active ulcer. Sensitive to changes in wound status. Appropriate for use in clinical trials of patients with DFU.	22; 25; 67
DFS-SF Diabetic Foot Ulcer Scale-Short Form [10,73]	Specific	29 questions in 6 subscales (Leisure, Physical health, Dependence/daily life, Negative emotions, Worries about ulcers/feet, Impact of ulcer care) Questions are graded on a 5-point Likert-type scale (1 = “not at all or none of the time”, 5 = “a lot, all of the time, or extremely”).	Score 0–100 (0 = poorer QoL)	Shortened and faster version of DFS. About 15 min to compile. Validated against DFS.	44; 50; 58; 59; 70
EQ-5D EuroQoL 5D Health utility Index [10,19,74]	Generic/ Utility	5 domains (Mobility, Self-care, Usual activities, Pain or discomfort, Anxiety or depression). Three (EQ-5D-3L) or five (EQ-5D-5L) possible answers for each domain. Accompanied by VAS analogue scale 0–100 (0 = worst imaginable health; 100 = best imaginable health) One additional question on current health state: is better, the same, or worse than your general health level over the past 12 months?	Scores for each response are summed using a formula that weights the different domains based on the EQ-5D scores of the general population. The generated numerical index ranges from −0.594 to 1. (Score 0 represent no QoL; <0 states perceived worse than death)	The 5 dimensions assessed can be transcribed into 243 possible health states. Intended as a research tool—not recommended for use in routine clinical practice. Used for the economic evaluation of foot care/treatment in diabetics.	19; 37; 42; 49; 51; 60; 69
FAAM Foot and Ankle Ability Measure [75]	Specific	2 sections ADL: 21 questions SPORT: 8 questions Each item is scored on a 5-point Likert scale (4 to 0) from “no difficulty at all” to “unable to perform”.	Item score totals, which range from 0 to 84 and 0 to 32 for the ADL and Sports subscales, respectively, were transformed to percentage scores. Higher scores represent a higher level of function for each subscale.	Region-specific, designed to assess lower limb function. Validated to measure physical function in patients with a broad spectrum of lower limb musculoskeletal disorders. Also responsive to lower limb function assessment in patients with diabetic foot. Most adapted in different languages and populations	40; 41; 43; 52; 53; 56
NeuroQoL [7,76]	Specific	35 questions 13 assess somatic experiences in 3 domains 14 assess specific functional, social and emotional experiences in 3 domains 1 assesses quality of life in each of the 6 domains 2 assess general satisfaction 1 asks the patient to express a specific judgment on his/her experience with podalic problems 1 asks for a general judgment on QoL Responses on a 5-point Likert scale from “never” to “all the time”	2 scores: physical symptoms and psychosocial function For each of the 27 specific questions, the patient is asked to judge the degree to which each experience was a nuisance or an important thing. The discomfort/importance has a score from 1 = not at all to 3 = very much The score is calculated by multiplying the Likert scale score with the corresponding discomfort/importance score. Higher score = worst QoL	Patients with peripheral neuropathy and diabetic foot ulcers. Poorly sensitive to lesion severity. 2 scores: physical symptoms and psychosocial function	46; 60
RAND-36 [17,77]	Generic	Assesses the same dimensions of the SF-36 and includes the same item on perceived change in health	Like SF-36 but with differences in scoring algorithm. It is possible to calculate two summary scores (physical health and mental health)	Adaptation of the SF-36 developed by the RAND Corporation	44; 68
SF-36 The medical Outcomes Study 36-item Short-Form Health Survey [10,78]	Generic	36 questions for 8 domains (Physical function, Bodily pain, General health perception, Vitality, Social functioning, Role limitations due to physical/emotional problems, Mental health).	3 scores: Overall HRQL Mental Component Summary Score (MCS-36) Physical Component Summary Score (PCS-36) Overall HRQL Score 0–100 (0 = poor HRQL)	Facilitates comparison with other chronic pathologies. Not specific for diabetic foot syndrome. Most frequently used.	20; 21; 22; 23; 25; 26; 27; 28; 29; 30; 31; 32; 33; 34; 35; 36; 40; 41; 43; 45; 47; 52; 53; 54; 55; 56; 62; 63; 66
SF-12 The medical Outcomes Study 12-item Short-Form Health Survey [10,79]	Generic	12 questions measuring the 8 domains of the SF-36.	Overall HRQL Score 0–100 (0 = poor HRQL). Two summary scores, PCS and MCS, can also be computed from the sub-scale scores.	Short and adapted version of the SF-36. Brevity lends its use for condition-specific investigations used in clinical trials. Can be converted to SF-6D and used for economic evaluations.	24
SF-8 The medical Outcomes Study 8-item Short-Form Health Survey [80]	Generic	8 questions measuring the 8 domains of the SF-36.	Overall HRQL Score 0–100 (0 = poor HRQL). Two summary scores, PCS and MCS, can also be computed from the sub-scale scores.	Short and adapted version of the SF-36. Derived from the SF-36 to minimize respondent burden. A valid tool for assessing HRQoL, especially in large-scale observational studies.	59
WHOQOL-BREF [9]	Generic	26 items in 4 domains: physical, psychological, social and environment Questions are graded on a 1–5 point Likert scale (1 = “disagree” or “not at all” and 5 = “totally agree” or “very”).	Domains are not scored where 20% of items or more are missing, and are unacceptable where two or more items are missed (or 1-item in the 3-item social domain). The scores are transformed on a scale 0–100	Assesses the individual health and well-being over the past two weeks. Short version of the WHOQOL-100.	8; 38; 39; 48; 57; 65
WoundQoL [7,81]	Specific	17 questions in 3 domains: 5 body 5 psyche 6 everyday life +1 question assessing financial burden Each question, 5 answers (score 0–4): not at all, a little, moderately, a lot and very much	Overall score calculated by adding all questions. It can only be computed if at least 75% of the items have been answered. Subscale scores calculated by adding individual questions. Higher score = Worst QoL	Patients with chronic wounds.	64

## Supplementary Materials

The following supporting information can be downloaded at: https://www.mdpi.com/article/10.3390/jcm14176116/s1, The file named ‘supplementary materials’ lists all the questionnaires taken into consideration in this review; it has also specified which ones are attached in pdf format and those that could not be found at the internet address where they could be consulted.
