# Supplementary material for: Patient-Reported Experience (PREMs) and Outcome (PROMs) Measures in Diabetic Foot Disease Management—A Scoping Review
Source: J Clin Med. 2025 Aug 29;14(17):6116. doi: 10.3390/jcm14176116 (PMC12429607; doi:10.3390/jcm14176116)
Supplement: Supplementary file 1 [file jcm-14-06116-s001.zip › S.M. 10 NEURO-QOL.pdf]

Communication – Short Form

Please respond to each question or statement by marking one box per row.

	**How much DIFFICULTY do you currently have…**	**None**	**A little**	**Somewhat**	**A lot**	**Cannot do**
NQCOG01	writing notes to yourself, such as appointments or 'to do' lists?............................	☐ 5	☐ 4	☐ 3	☐ 2	☐ 1
NQCOG04	understanding family and friends on the phone?...	☐ 5	☐ 4	☐ 3	☐ 2	☐ 1
NQCOG08	carrying on a conversation with a small group of familiar people (e.g., family or a few friends)?..	☐ 5	☐ 4	☐ 3	☐ 2	☐ 1
NQCOG10	organizing what you want to say?...................	☐ 5	☐ 4	☐ 3	☐ 2	☐ 1
NQCOG11	speaking clearly enough to use the telephone?..	☐ 5	☐ 4	☐ 3	☐ 2	☐ 1

©2008-2013 David Cella and the PROMIS Health Organization on behalf of the National Institute for Neurological Disorders and Stroke (NINDS). Used with permission.

Ability to Participate in Social Roles and Activities – Short Form

Please respond to each question or statement by marking one box per row.

	**In the past 7 days…**	**Never**	**Rarely**	**Sometimes**	**Often**	**Always**
NQPRF01	I can keep up with my family responsibilities……………………………	☐ 1	☐ 2	☐ 3	☐ 4	☐ 5
NQPRF03	I am able to do all of my regular family activities………………………………….	☐ 1	☐ 2	☐ 3	☐ 4	☐ 5
NQPRF08	I am able to socialize with my friends…...	☐ 1	☐ 2	☐ 3	☐ 4	☐ 5
NQPRF09	I am able to do all of my regular activities with friends……………………………….	☐ 1	☐ 2	☐ 3	☐ 4	☐ 5
NQPRF17	I can keep up with my social commitments……………………………..	☐ 1	☐ 2	☐ 3	☐ 4	☐ 5
NQPRF26	I am able to participate in leisure activities………………………………….	☐ 1	☐ 2	☐ 3	☐ 4	☐ 5
NQPRF32	I am able to perform my daily routines…..	☐ 1	☐ 2	☐ 3	☐ 4	☐ 5
NQPRF34	I can keep up with my work responsibilities (include work at home)….	☐ 1	☐ 2	☐ 3	☐ 4	☐ 5

©2008-2013 David Cella and the PROMIS Health Organization on behalf of the National Institute for Neurological Disorders and Stroke (NINDS). Used with permission.

Anxiety – Short Form

Please respond to each question or statement by marking one box per row.

	**In the past 7 days…**	**Never**	**Rarely**	**Sometimes**	**Often**	**Always**
NQANX26	I felt uneasy………………………………	☐ 1	☐ 2	☐ 3	☐ 4	☐ 5
NQANX22	I felt nervous…………………………...	☐ 1	☐ 2	☐ 3	☐ 4	☐ 5
NQANX23	Many situations made me worry…………	☐ 1	☐ 2	☐ 3	☐ 4	☐ 5
NQANX20	My worries overwhelmed me…………….	☐ 1	☐ 2	☐ 3	☐ 4	☐ 5
NQANX27	I felt tense……………………………...	☐ 1	☐ 2	☐ 3	☐ 4	☐ 5
NQANX28	I had difficulty calming down….………...	☐ 1	☐ 2	☐ 3	☐ 4	☐ 5
NQANX09	I had sudden feelings of panic……………	☐ 1	☐ 2	☐ 3	☐ 4	☐ 5
NQANX07	I felt nervous when my normal routine was disturbed……………………….…….	☐ 1	☐ 2	☐ 3	☐ 4	☐ 5

©2008-2013 David Cella and the PROMIS Health Organization on behalf of the National Institute for Neurological Disorders and Stroke (NINDS). Used with permission.

Depression – Short Form

Please respond to each question or statement by marking one box per row.

	In the past 7 days…	Never	Rarely	Sometimes	Often	Always
NQDEP13	I felt depressed…………………………..	☐ 1	☐ 2	☐ 3	☐ 4	☐ 5
NQDEP23	I felt hopeless…………………………….	☐ 1	☐ 2	☐ 3	☐ 4	☐ 5
NQDEP07	I felt that nothing could cheer me up……..	☐ 1	☐ 2	☐ 3	☐ 4	☐ 5
NQDEP27	I felt that my life was empty……………..	☐ 1	☐ 2	☐ 3	☐ 4	☐ 5
NQDEP02	I felt worthless……………………………	☐ 1	☐ 2	☐ 3	☐ 4	☐ 5
NQDEP19	I felt unhappy…………………………….	☐ 1	☐ 2	☐ 3	☐ 4	☐ 5
NQDEP21	I felt I had no reason for living…………..	☐ 1	☐ 2	☐ 3	☐ 4	☐ 5
NQDEP24	I felt that nothing was interesting………..	☐ 1	☐ 2	☐ 3	☐ 4	☐ 5

©2008-2013 David Cella and the PROMIS Health Organization on behalf of the National Institute for Neurological Disorders and Stroke (NINDS). Used with permission.

Emotional and Behavioral Dyscontrol – Short Form

Please respond to each question or statement by marking one box per row.

	**In the past 7 days…**	**Never**	**Rarely**	**Sometimes**	**Often**	**Always**
NQPER02	I had trouble controlling my temper……...	☐ 1	☐ 2	☐ 3	☐ 4	☐ 5
NQPER05	It was hard to control my behavior……….	☐ 1	☐ 2	☐ 3	☐ 4	☐ 5
NQPER06	I said or did things without thinking……..	☐ 1	☐ 2	☐ 3	☐ 4	☐ 5
NQPER07	I got impatient with other people………...	☐ 1	☐ 2	☐ 3	☐ 4	☐ 5
NQPER11	I was irritable around other people……….	☐ 1	☐ 2	☐ 3	☐ 4	☐ 5
NQPER12	I was bothered by little things……………	☐ 1	☐ 2	☐ 3	☐ 4	☐ 5
NQPER17	I became easily upset……………………..	☐ 1	☐ 2	☐ 3	☐ 4	☐ 5
NQPER19	I was in conflict with others……………...	☐ 1	☐ 2	☐ 3	☐ 4	☐ 5

©2008-2013 David Cella and the PROMIS Health Organization on behalf of the National Institute for Neurological Disorders and Stroke (NINDS). Used with permission.

Fatigue – Short Form

Please respond to each question or statement by marking one box per row.

	In the past 7 days…	Never	Rarely	Sometimes	Often	Always
NQFTG13	I felt exhausted…………………………...	☐ 1	☐ 2	☐ 3	☐ 4	☐ 5
NQFTG11	I felt that I had no energy………………...	☐ 1	☐ 2	☐ 3	☐ 4	☐ 5
NQFTG15	I felt fatigued……………………………..	☐ 1	☐ 2	☐ 3	☐ 4	☐ 5
NQFTG06	I was too tired to do my household chores.	☐ 1	☐ 2	☐ 3	☐ 4	☐ 5
NQFTG07	I was too tired to leave the house………...	☐ 1	☐ 2	☐ 3	☐ 4	☐ 5
NQFTG10	I was frustrated by being too tired to do the things I wanted to do…………………	☐ 1	☐ 2	☐ 3	☐ 4	☐ 5
NQFTG14	I felt tired…………………………………	☐ 1	☐ 2	☐ 3	☐ 4	☐ 5
NQFTG02	I had to limit my social activity because I was tired…………………………………	☐ 1	☐ 2	☐ 3	☐ 4	☐ 5

©2008-2013 David Cella and the PROMIS Health Organization on behalf of the National Institute for Neurological Disorders and Stroke (NINDS). Used with permission.

Lower Extremity Function (Mobility) – Short Form

Please respond to each question or statement by marking one box per row.

		Without any difficulty	With a little difficulty	With some difficulty	With much difficulty	Unable to do
NQMOB37	Are you able to get on and off the toilet?...	☐ 5	☐ 4	☐ 3	☐ 2	☐ 1
NQMOB30	Are you able to step up and down curbs?...	☐ 5	☐ 4	☐ 3	☐ 2	☐ 1
NQMOB26	Are you able to get in and out of a car?.....	☐ 5	☐ 4	☐ 3	☐ 2	☐ 1
NQMOB32	Are you able to get out of bed into a chair?...	☐ 5	☐ 4	☐ 3	☐ 2	☐ 1
NQMOB25	Are you able to push open a heavy door?..	☐ 5	☐ 4	☐ 3	☐ 2	☐ 1
NQMOB33	Are you able to run errands and shop?.......	☐ 5	☐ 4	☐ 3	☐ 2	☐ 1
NQMOB31	Are you able to get up off the floor from lying on your back without help?..............	☐ 5	☐ 4	☐ 3	☐ 2	☐ 1
NQMOB28	Are you able to go for a walk of at least 15 minutes?...	☐ 5	☐ 4	☐ 3	☐ 2	☐ 1

©2008-2013 David Cella and the PROMIS Health Organization on behalf of the National Institute for Neurological Disorders and Stroke (NINDS). Used with permission.

Positive Affect and Well-Being - Short Form

Please respond to each question or statement by marking one box per row.

	**Lately…**	**Never**	**Rarely**	**Sometimes**	**Often**	**Always**
NQPPF14	I had a sense of well-being………………	☐ 1	☐ 2	☐ 3	☐ 4	☐ 5
NQPPF12	I felt hopeful……………………………..	☐ 1	☐ 2	☐ 3	☐ 4	☐ 5
NQPPF15	My life was satisfying……………………	☐ 1	☐ 2	☐ 3	☐ 4	☐ 5
NQPPF20	My life had purpose………………………	☐ 1	☐ 2	☐ 3	☐ 4	☐ 5
NQPPF17	My life had meaning……………………..	☐ 1	☐ 2	☐ 3	☐ 4	☐ 5
NQPPF22	I felt cheerful……………………………..	☐ 1	☐ 2	☐ 3	☐ 4	☐ 5
NQPPF19	My life was worth living…………………	☐ 1	☐ 2	☐ 3	☐ 4	☐ 5
NQPPF16	I had a sense of balance in my life………	☐ 1	☐ 2	☐ 3	☐ 4	☐ 5
NQPPF07	Many areas of my life were interesting to me……………………………………...	☐ 1	☐ 2	☐ 3	☐ 4	☐ 5

©2008-2013 David Cella and the PROMIS Health Organization on behalf of the National Institute for Neurological Disorders and Stroke (NINDS). Used with permission.

Sleep Disturbance – Short Form

Please respond to each question or statement by marking one box per row.

	**In the past 7 days…**	**Never**	**Rarely**	**Sometimes**	**Often**	**Always**
NQSLP02	I had to force myself to get up in the morning…………………………………..	☐ 1	☐ 2	☐ 3	☐ 4	☐ 5
NQSLP03	I had trouble stopping my thoughts at bedtime…………………………………....	☐ 1	☐ 2	☐ 3	☐ 4	☐ 5
NQSLP04	I was sleepy during the daytime…………	☐ 1	☐ 2	☐ 3	☐ 4	☐ 5
NQSLP05	I had trouble sleeping because of bad dreams…………………………………..	☐ 1	☐ 2	☐ 3	☐ 4	☐ 5
NQSLP07	I had trouble falling asleep………………	☐ 1	☐ 2	☐ 3	☐ 4	☐ 5
NQSLP12	Pain woke me up…………………………	☐ 1	☐ 2	☐ 3	☐ 4	☐ 5
NQSLP13	I avoided or cancelled activities with my friends because I was tired from having a bad night's sleep…………………………	☐ 1	☐ 2	☐ 3	☐ 4	☐ 5
NQSLP18	I felt physically tense during the middle of the night or early morning hours………..	☐ 1	☐ 2	☐ 3	☐ 4	☐ 5

©2008-2013 David Cella and the PROMIS Health Organization on behalf of the National Institute for Neurological Disorders and Stroke (NINDS). Used with permission.

Upper Extremity Function (Fine Motor, ADL) – Short Form

Please respond to each question or statement by marking one box per row.

		Without any difficulty	With a little difficulty	With some difficulty	With much difficulty	Unable to do
NQUEX29	Are you able to turn a key in a lock?..........	☐ 5	☐ 4	☐ 3	☐ 2	☐ 1
NQUEX20	Are you able to brush your teeth?.............	☐ 5	☐ 4	☐ 3	☐ 2	☐ 1
NQUEX44	Are you able to make a phone call using a touch tone key-pad?..................................	☐ 5	☐ 4	☐ 3	☐ 2	☐ 1
NQUEX36	Are you able to pick up coins from a table top?...	☐ 5	☐ 4	☐ 3	☐ 2	☐ 1
NQUEX30	Are you able to write with a pen or pencil?..	☐ 5	☐ 4	☐ 3	☐ 2	☐ 1
NQUEX28	Are you able to open and close a zipper?...	☐ 5	☐ 4	☐ 3	☐ 2	☐ 1
NQUEX33	Are you able to wash and dry your body?..	☐ 5	☐ 4	☐ 3	☐ 2	☐ 1
NQUEX37	Are you able to shampoo your hair?..........	☐ 5	☐ 4	☐ 3	☐ 2	☐ 1

©2008-2013 David Cella and the PROMIS Health Organization on behalf of the National Institute for Neurological Disorders and Stroke (NINDS). Used with permission.

Stigma-Short Form

Please respond to each question or statement by marking one box per row.

	**Lately…**	**Never**	**Rarely**	**Sometimes**	**Often**	**Always**
NQSTG02	Because of my illness, some people avoided me………………………………	☐ 1	☐ 2	☐ 3	☐ 4	☐ 5
NQSTG04	Because of my illness, I felt left out of things……………………………………	☐ 1	☐ 2	☐ 3	☐ 4	☐ 5
NQSTG 08	Because of my illness, people avoided looking at me……………………………	☐ 1	☐ 2	☐ 3	☐ 4	☐ 5
NQSTG 16	I felt embarrassed about my illness……..	☐ 1	☐ 2	☐ 3	☐ 4	☐ 5
NQSTG 01	Because of my illness, some people seemed uncomfortable with me………..	☐ 1	☐ 2	☐ 3	☐ 4	☐ 5
NQSTG 17	I felt embarrassed because of my physical limitations….……………………………	☐ 1	☐ 2	☐ 3	☐ 4	☐ 5
NQSTG05	Because of my illness, people were unkind to me …………………………….	☐ 1	☐ 2	☐ 3	☐ 4	☐ 5
NQSTG21	Some people acted as though it was my fault I have this illness..…………………	☐ 1	☐ 2	☐ 3	☐ 4	☐ 5

©2008-2013 David Cella and the PROMIS Health Organization on behalf of the National Institute for Neurological Disorders and Stroke (NINDS). Used with permission.

Satisfaction with Social Roles and Activities – Short Form

Please respond to each question or statement by marking one box per row.

	In the past 7 days…	Not at all	A little bit	Somewhat	Quite a bit	Very much
NQSAT 03	I am bothered by my limitations in regular family activities	☐ 5	☐ 4	☐ 3	☐ 2	☐ 1
NQSAT 23	I am disappointed in my ability to socialize with my family………………...	☐ 5	☐ 4	☐ 3	☐ 2	☐ 1
NQSAT14	I am bothered by limitations in my regular activities with friends……………………	☐ 5	☐ 4	☐ 3	☐ 2	☐ 1
NQSAT11	I am disappointed in my ability to meet the needs of my friends	☐ 5	☐ 4	☐ 3	☐ 2	☐ 1

	In the past 7 days…	Not at all	A little bit	Somewhat	Quite a bit	Very much
NQSAT33	I am satisfied with my ability to do things for fun outside my home………………....	☐ 1	☐ 2	☐ 3	☐ 4	☐ 5
NQSAT32	I am satisfied with the amount of time I spend doing leisure activities…………….	☐ 1	☐ 2	☐ 3	☐ 4	☐ 5
NQSAT47	I am satisfied with how much of my work I can do (include work at home)…………	☐ 1	☐ 2	☐ 3	☐ 4	☐ 5
NQSAT 46	I am satisfied with my ability to do household chores or tasks………..........	☐ 1	☐ 2	☐ 3	☐ 4	☐ 5

©2008-2013 David Cella and the PROMIS Health Organization on behalf of the National Institute for Neurological Disorders and Stroke (NINDS). Used with permission.

Cognition Function– Short Form

Please respond to each question or statement by marking one box per row.

	**In the past 7 days…**	**Never**	**Rarely (once)**	**Sometimes (2-3 times)**	**Often (once a day)**	**Very often (several times a day)**
NQCOG64	I had to read something several times to understand it……………………………..	☐ 5	☐ 4	☐ 3	☐ 2	☐ 1
NQCOG75	My thinking was slow……………………	☐ 5	☐ 4	☐ 3	☐ 2	☐ 1
NQCOG77	I had to work really hard to pay attention or I would make a mistake………………	☐ 5	☐ 4	☐ 3	☐ 2	☐ 1
NQCOG80	I had trouble concentrating………………	☐ 5	☐ 4	☐ 3	☐ 2	☐ 1

	**How much DIFFICULTY do you currently have…**	**None**	**A little**	**Somewhat**	**A lot**	**Cannot do**
NQCOG22	reading and following complex instructions (e.g., directions for a new medication)?..	☐ 5	☐ 4	☐ 3	☐ 2	☐ 1
NQCOG24	planning for and keeping appointments that are not part of your weekly routine, (e.g., a therapy or doctor appointment, or a social gathering with friends and family)?..	☐ 5	☐ 4	☐ 3	☐ 2	☐ 1
NQCOG25	managing your time to do most of your daily activities?...	☐ 5	☐ 4	☐ 3	☐ 2	☐ 1
NQCOG40	learning new tasks or instructions?...........	☐ 5	☐ 4	☐ 3	☐ 2	☐ 1

©2008-2013 David Cella and the PROMIS Health Organization on behalf of the National Institute for Neurological Disorders and Stroke (NINDS). Used with permission.
