# Supplementary material for: Patient-Reported Experience (PREMs) and Outcome (PROMs) Measures in Diabetic Foot Disease Management—A Scoping Review
Source: J Clin Med. 2025 Aug 29;14(17):6116. doi: 10.3390/jcm14176116 (PMC12429607; doi:10.3390/jcm14176116)
Supplement: Supplementary file 1 [file jcm-14-06116-s001.zip › S.M. 3 SF-8.pdf]

SF 8 Questionnaire

1. Overall, how would you rate your health during the past 4 weeks?

Excellent ☐ Very good ☐ Good ☐ Fair ☐ Poor ☐ Very poor ☐

2. During the past 4 weeks, how much did physical health problems limit your usual physical activities (such as walking or climbing stairs)?

Not at all ☐ Very little ☐ Somewhat ☐ Quite a lot ☐ Could not do physical activities ☐

3. During the past 4 weeks, how much difficulty did you have doing your daily work, both at home and away from home, because of your physical health?

None at all ☐ A little bit ☐ Some ☐ Quite a lot ☐ Could not do daily work ☐

4. How much bodily pain have you had during the past 4 weeks?

None ☐ Very mild ☐ Mild ☐ Moderate ☐ Severe ☐ Very Severe ☐

5. During the past 4 weeks, how much energy did you have?

Very much ☐ Quite a lot ☐ Some ☐ A little ☐ None ☐

6. During the past 4 weeks, how much did your physical health or emotional problems limit your usual social activities with family or friends?

Not at all ☐ Very little ☐ Somewhat ☐ Quite a lot ☐ Could not do social activities ☐

7. During the past 4 weeks, how much have you been bothered by emotional problems (such as feeling anxious, depressed or irritable)?

Not at all ☐ Slightly ☐ Moderately ☐ Quite a lot ☐ Extremely ☐

8. During the past 4 weeks, how much did personal or emotional problems keep you from doing your usual work, school or other daily activities?

Not at all ☐ Very little ☐ Somewhat ☐ Quite a lot ☐ Could not do daily activities ☐

Thank you for completing these questions!
