# Supplementary material for: Patient-Reported Experience (PREMs) and Outcome (PROMs) Measures in Diabetic Foot Disease Management—A Scoping Review
Source: J Clin Med. 2025 Aug 29;14(17):6116. doi: 10.3390/jcm14176116 (PMC12429607; doi:10.3390/jcm14176116)
Supplement: Supplementary file 1 [file jcm-14-06116-s001.zip › S.M. 6 CWIS.pdf]

Overall Quality of Life

We would like you to rate your overall quality of life during the past 7 days.

Please circle a number below

How good is your quality of life?

My quality of life is the worst possible	0	1	2	3	4	5	6	7	8	9	10	My quality of life is the best possible

How satisfied are you with your overall quality of life?

Not at all satisfied	0	1	2	3	4	5	6	7	8	9	10	Very satisfied

Overall Comments

© Copyright WHRU 1997

For Office Use

Total

Wound Healing Research Unit

University of Wales College of Medicine

Cardiff Wound Impact Questionnaire

© Copyright WHRU 1997

The following questionnaire is concerned with the effects that your wound(s) has (have) on your daily life. Please answer the questions carefully by placing a check mark in the box which most closely reflects how you feel; it should take about ten minutes to complete.

If you are unsure about how to answer a question, please mark the answer which is closest to how you feel. All answers are confidential.

Personal Details

Patient Initials ● ● ● **Sex** M ● F ●

Patient Number ● ● ● ●

Date of Birth M M ● ● D D ● ● Y Y ● ●

	1st	2nd	3rd	4th	5th
**Assessment**	☐	☐	☐	☐	☐

Assessment Date
M M ● ● D D ● ● Y Y ● ●

Next Assessment Due
M M ● ● D D ● ● Y Y ● ●

Wound(s) status Healed ☐ Not Healed ☐

Do you live on your own? Yes ☐ No ☐

How often do you see your family and friends?

Daily ☐ Once a month ☐

© Copyright WHRU 1997
f:\institut\cultadap\project\jj1797\final versions\cwisusaq.doc – 28/02/2003

For Office Use **Total** ☐

Once a week ☐ Less than once a month ☐

© Copyright WHRU 1997
f:\institut\cultadap\project\jj1797\final versions\cwisusaq.doc – 28/02/2003

For Office Use **Total** ☐

Social Life

How stressful has this experience been for you during the past 7 days?

	Not at all/ Not applicable	Slightly	Moderately	Quite a bit	Very
Difficulty getting out and around	☐	☐	☐	☐	☐
Relying more on others	☐	☐	☐	☐	☐
Your family/friends being overly protective	☐	☐	☐	☐	☐
Unable to enjoy your usual social life (eg hobbies)	☐	☐	☐	☐	☐
Limited contact with family/friends	☐	☐	☐	☐	☐
Not going out for fear of bumping your wound site	☐	☐	☐	☐	☐
Wanting to withdraw from people	☐	☐	☐	☐	☐

© Copyright WHRU 1997

For Office Use **Total** ☐

Social Life

Have you experienced any of the following during the past 7 days?

	Not at all/ Not applicable	Seldom	Sometimes	Frequently	Always
Difficulty getting out and around	☐	☐	☐	☐	☐
Relying more on others	☐	☐	☐	☐	☐
Your family/friends being overly protective	☐	☐	☐	☐	☐
Unable to enjoy your usual social life (eg hobbies)	☐	☐	☐	☐	☐
Limited contact with family/friends	☐	☐	☐	☐	☐
Not going out for fear of bumping your wound site	☐	☐	☐	☐	☐
Wanting to withdraw from people	☐	☐	☐	☐	☐

© Copyright WHRU 1997
f:\institut\cultadap\project\jj1797\final versions\cwisusaq.doc – 28/02/2003

For Office Use **Total** ☐

Well-being

To what extent do you agree/disagree with the following statements?

	Strongly Disagree	Disagree	Not Sure	Agree	Strongly Agree
I feel anxious about my wound(s)	☐	☐	☐	☐	☐
I feel frustrated with the time it is taking for the wound(s) to heal	☐	☐	☐	☐	☐
I am confident that the wound(s) I have will heal	☐	☐	☐	☐	☐
I worry that I may get another wound in the future	☐	☐	☐	☐	☐
The appearance of the wound site is upsetting to me	☐	☐	☐	☐	☐
I worry about bumping the wound site	☐	☐	☐	☐	☐
I worry about the impact of the wound(s) on my family/friends	☐	☐	☐	☐	☐

For Office Use **Total** ☐

© Copyright WHRU 1997

Physical Symptoms and Daily Living

Have you <u>experienced</u> any of the following during the past 7 days?

	Not at all/ Not applicable	Seldom	Sometimes	Frequently	Always
Disturbed sleep	☐	☐	☐	☐	☐
Difficulty bathing	☐	☐	☐	☐	☐
Immobility around the home	☐	☐	☐	☐	☐
Immobility outside the home	☐	☐	☐	☐	☐
Leakage from the wound(s)	☐	☐	☐	☐	☐
Pain from the wound site	☐	☐	☐	☐	☐
Discomfort from the bandaging/dressing	☐	☐	☐	☐	☐
Unpleasant odor or smell from the wound(s)	☐	☐	☐	☐	☐
Problems with everyday tasks (eg shopping)	☐	☐	☐	☐	☐
Difficulty in finding appropriate footwear	☐	☐	☐	☐	☐
Problems with the amount of time needed to care for the wound site	☐	☐	☐	☐	☐
Financial difficulties as a result of the wound(s)	☐	☐	☐	☐	☐

For Office Use **Total** ☐

© Copyright WHRU 1997

Physical Symptoms and Daily Living

How <u>stressful</u> has this experience been for you during the past 7 days?

	Not at all/ Not applicable	Slightly	Moderately	Quite a bit	Very
Disturbed sleep	☐	☐	☐	☐	☐
Difficulty bathing	☐	☐	☐	☐	☐
Immobility around the home	☐	☐	☐	☐	☐
Immobility outside the home	☐	☐	☐	☐	☐
Leakage from the wound(s)	☐	☐	☐	☐	☐
Pain from the wound site	☐	☐	☐	☐	☐
Discomfort from the bandaging/dressing	☐	☐	☐	☐	☐
Unpleasant odor or smell from the wound(s)	☐	☐	☐	☐	☐
Problems with everyday tasks (eg shopping)	☐	☐	☐	☐	☐
Difficulty in finding appropriate footwear	☐	☐	☐	☐	☐
Problems with the amount of time needed to care for the wound site	☐	☐	☐	☐	☐
Financial difficulties as a result of the wound(s)	☐	☐	☐	☐	☐

For Office Use **Total** ☐

© Copyright WHRU 1997

© Copyright WHRU 1997
f:\institut\cultadap\project\jj1797\final versions\cwisusaq.doc – 28/02/2003
For Office Use **Total**
