# Supplementary material for: Patient-Reported Experience (PREMs) and Outcome (PROMs) Measures in Diabetic Foot Disease Management—A Scoping Review
Source: J Clin Med. 2025 Aug 29;14(17):6116. doi: 10.3390/jcm14176116 (PMC12429607; doi:10.3390/jcm14176116)
Supplement: Supplementary file 1 [file jcm-14-06116-s001.zip › S.M. 8 WOUND-QOL-17.pdf]

Wound-QoL-17 questionnaire on quality of life with chronic wounds

With the following questions, we aim to find out how your chronic wound(s) affect(s) your quality of life.

Please tick one box per line!

**In the <u>last seven days</u> …**		**not at all**	**a little**	**moderately**	**quite a lot**	**very much**
1	…my wound hurt	O	O	O	O	O
2	…my wound had a bad smell	O	O	O	O	O
3	…there was a disturbing discharge from the wound	O	O	O	O	O
4	…the wound has affected my sleep	O	O	O	O	O
5	…the treatment of the wound has been a burden to me	O	O	O	O	O
6	…the wound has made me unhappy	O	O	O	O	O
7	…I have felt frustrated because the wound is taking so long to heal	O	O	O	O	O
8	…I have worried about my wound	O	O	O	O	O
9	…I have been afraid of the wound getting worse or of new wounds appearing	O	O	O	O	O
10	…I have been afraid of knocking the wound	O	O	O	O	O
11	…I have had trouble moving about because of the wound	O	O	O	O	O
12	…climbing stairs has been difficult because of the wound	O	O	O	O	O
13	…I have had trouble with day-to-day activities because of the wound	O	O	O	O	O
14	…the wound has limited my leisure activities	O	O	O	O	O
15	…the wound has forced me to limit my activities with others	O	O	O	O	O
16	…I have felt dependent on help from others because of the wound	O	O	O	O	O
17	…the wound has been a financial burden to me	O	O	O	O	O
