# Supplementary material for: Patient-Reported Experience (PREMs) and Outcome (PROMs) Measures in Diabetic Foot Disease Management—A Scoping Review
Source: J Clin Med. 2025 Aug 29;14(17):6116. doi: 10.3390/jcm14176116 (PMC12429607; doi:10.3390/jcm14176116)
Supplement: Supplementary file 1 [file jcm-14-06116-s001.zip › Supplemental Materials.pdf]

Supplemental Materials

Questionnaires available in pdf format

- **EQ-5D-5L** named *S.M. 1 EQ-5D-5L*
- **EQ-5D-3L** named *S.M. 2 EQ-5D-3L*
- **SF-8** named *S.M. 3 SF-8*
- **SF-12** named *S.M. 4 SF-12*
- **SF-36/RAND 36** named *S.M. 5 WOUND-QOL-17*
- **CWIS** named *S.M. 6 CWIS*
- **WHOQOL-BREF** named *S.M. 7 QHOQOL-BREF*
- **WOUND-QOL-17** named *S.M. 8 WOUND-QOL-17*
- **FAAM** named *S.M. 9 FAAM*
- **NEURO-QOL** named *S.M. 10 NEURO-QOL*

Questionnaires not available in pdf but downloadable at the following addresses

- **DFS:** https://eprovide.mapi-trust.org/instruments/diabetic-foot-ulcer-scale-short-form
- **DFS-SF:** https://eprovide.mapi-trust.org/instruments/diabetic-foot-ulcer-scale-short-form
